# Epidemiological trends in notified influenza cases in Australia’s Northern Territory, 2007‐2016

**DOI:** 10.1111/irv.12757

**Published:** 2020-05-23

**Authors:** Aaron L. Weinman, Sheena G. Sullivan, Dhanasekaran Vijaykrishna, Peter Markey, Avram Levy, Adrian Miller, Steven Y. C. Tong

**Affiliations:** ^1^ Doherty Department Peter Doherty Institute for Infection and Immunity University of Melbourne Melbourne Victoria Australia; ^2^ Doherty Department WHO Collaborating Centre for Reference and Research on Influenza Royal Melbourne Hospital Peter Doherty Institute for Infection and Immunity University of Melbourne Melbourne Victoria Australia; ^3^ Department of Microbiology Biomedicine Discovery Institute Monash University Clayton Victoria Australia; ^4^ WHO Collaborating Centre for Reference and Research on Influenza Royal Melbourne Hospital Peter Doherty Institute for Infection and Immunity Melbourne Victoria Australia; ^5^ Northern Territory Centre for Disease Control Casuarina Northern Territory Australia; ^6^ PathWest Laboratory Medicine Nedlands Western Australia Australia; ^7^ Centre for Indigenous Health and Equity Research CQUniversity Townsville Queensland Australia; ^8^ Doherty Department Victorian Infectious Diseases Service The Royal Melbourne Hospital Peter Doherty Institute for Infection and Immunity University of Melbourne Melbourne Victoria Australia; ^9^ Menzies School of Health Research Darwin Northern Territory Australia

**Keywords:** epidemics, epidemiology, influenza, Northern Territory

## Abstract

**Background:**

The Northern Territory (NT) of Australia has a mix of climates, sparsely distributed population and a large proportion of the populace are Indigenous Australians, and influenza is known to have a disproportionate impact upon this group. Understanding the epidemiology of influenza in this region would inform public health strategies.

**Objectives:**

To assess if there are consistent patterns in characteristics of influenza outbreaks in the NT.

**Methods:**

Laboratory confirmed influenza cases in the NT are notified to the NT Centre for Disease Control. We conducted analyses on notified cases from 2007‐2016 to determine incidence rates (by age group, Indigenous status and area), seasonality of cases and spatial distribution of influenza types. Notified cases were linked to laboratory datasets to update information on influenza type or subtype

**Results:**

The disparity in Indigenous and non‐Indigenous notification rates varied by age group, with rate ratios for Indigenous versus non‐Indigenous ranging from 1.58 (95% CI:1.39, 1.80) for ages 15‐24 to 5.56 (95% CI: 4.71, 6.57) for ages 55‐64. The disparity between Indigenous and non‐Indigenous notification rates appeared higher in the Central Australia region. Indigenous versus non‐Indigenous hospitalisation and mortality rate ratios were 6.51 (95% CI: 5.91, 7.18) and 5.46 (95% CI: 2.40, 12.71) respectively. Inter‐seasonal peaks during February and March occurred in 2011, 2013 and 2014, and were due to influenza activity in the tropical north of the NT.

**Conclusions:**

Our results highlight the importance of influenza vaccination across all age groups for Indigenous Australians. An early vaccination campaign targeted against outbreaks in February‐March would be best focused on the tropical north.

## 1 INTRODUCTION

Seasonal epidemics of the influenza virus represent a continual global challenge. These epidemics usually occur in the winter months of temperate regions, but in tropical regions, influenza is reported to circulate widely outside this season.^1^ Particular groups in the population, such as the elderly and pregnant women, are known to be at higher risk of severe outcomes from influenza infection.^2^ The virus has also caused the breakout of pandemics, the most recent being the 2009 H1N1 pandemic.^2^

Australia's Northern Territory has several features which are likely to make the epidemiology of influenza in this area unique. First, climatic conditions are variable across the Northern Territory. The northern section of the Northern Territory (known as the “Top End”) has a tropical climate, whereas the southern half of the Northern Territory (known as the “Central Australia” region) has a desert climate. The potential for circulation of the virus outside the typical influenza season in this tropical region could impact upon the optimal time for vaccination in this area.

Second, 30% of the Northern Territory’s population are Indigenous Australians, making the proportion of Indigenous Australians living in the Northern Territory over 5 times higher than that of any other jurisdiction in Australia.^3^ Influenza has had a particularly severe impact on Indigenous Australians. For example, serosurveys conducted to determine rates of A(H1N1)pdm09 in the Northern Territory estimated that 22.9% of the Indigenous population in the region experienced A(H1N1)pdm09 infection in 2009, compared to only 12.4% of the non‐Indigenous population.^4^ Furthermore, hospital admissions for A(H1N1)pdm09 in the Top End region were 12 times higher for Indigenous compared to non‐Indigenous Australians.^5^

Given the increased rates of influenza infection and severe outcomes from infection amongst the Indigenous population, beginning in 1999, the Australian Government funded influenza vaccination for Indigenous Australians aged 50 and older and those 15 and older that had a comorbid medical condition.^6^ Funded vaccination was expanded in 2010 to include all Indigenous Australians aged 15 and older, and expanded again in 2015 to include Indigenous Australians aged 6 months to under 5 years.^6^ Due to the disproportionate impact influenza has had upon Indigenous Australians and the large Indigenous population in the region, it is important that public health measures to counter the spread of influenza in the Northern Territory be optimised to mitigate the impact of future outbreaks. Previous surveillance of influenza activity in the Northern Territory has highlighted increased rates of influenza cases and severe outcomes of infection in the Indigenous population.^7^ However, the full impact of influenza, through pandemic and non‐pandemic periods, in the Indigenous and non‐Indigenous population has not been quantified. There also has not been a long‐term post‐pandemic comparison of the areas in which the disparity in rates of influenza is greatest. There have been reports of outbreaks of influenza activity in the Top End region of the Northern Territory outside the typical influenza season,^8^ but the regions of the Northern Territory these outbreaks affect have not been assessed in all years. Year‐by‐year analyses of the divisions of the Northern Territory in which influenza rates are highest are also lacking, as are comparisons of the distribution of influenza cases between the major towns and remote areas. A better understanding of the epidemiology of influenza in this region could lead to the development of more optimally targeted vaccination campaigns. In this study, laboratory‐confirmed influenza notifications were assessed to understand whether there are consistent patterns in the timing, geographic characteristics and groups affected by influenza in the Northern Territory.

## 2 MATERIALS AND METHODS

### 2.1 Data source

In the Northern Territory, laboratory‐confirmed influenza cases are notified to the Northern Territory Centre for Disease Control. The Centre for Disease Control collects meta‐data for each case including details about the patient’s age, sex, Indigenous status, residential location, influenza type, diagnosis date, hospitalisation status, dates of hospital stays (for hospitalised cases) and mortality. We analysed notifications from 2007 to 2016.

Following laboratory confirmation of influenza infection by pathology providers, further analysis of samples is carried out by PathWest Laboratory Medicine, Perth, or the WHO Collaborating Centre for Reference and Research on Influenza, Melbourne. Results of typing and subtyping performed by these groups on samples corresponding to notified cases were accessed.

Population denominator data were obtained from the Australian Bureau of Statistics.

### 2.2 Data cleaning and analysis

Duplicate notifications were deleted. Notifications for patients who resided outside the Northern Territory were excluded from the calculations of rates but included in analyses that used raw count data. Hospital admissions that occurred >14 days after or more than 3 days before influenza diagnosis were considered not to be influenza associated. Notifications missing hospitalisation dates were excluded from analysis of hospitalisations.

The location of each patient was classified into 2016 Statistical Area 2 (SA2) level groupings, as defined by the Australian Bureau of Statistics,^9^ based on the 2016 Australian Statistical Geography Standard Coding Indexes^10^ where possible. Locations of notified cases not found on the coding index were assigned manually using interactive maps supplied by the Australian Bureau of Statistics^11^ and the Northern Territory Places Names Register.^12^ SA2 areas that were recognised to form the suburban areas of the major cities of Darwin and Alice Springs were combined to form the Darwin and Alice Springs regions in analyses.

The notification data set was linked to corresponding patient samples stored at PathWest Laboratory Medicine, Perth, or the WHO Collaborating Centre for Reference and Research on Influenza, Melbourne. Notification data were linked to samples by matching sample date and at least one of the fields of patient name and date of birth. Information present on the databases of these laboratories was used to update information on influenza type or subtype in the notifications data set. For analyses of spatial distribution and seasonality, cases of co‐infections with type A and B were counted as separate notifications for each type.

Case notification rates in each age group in the Northern Territory by Indigenous status and age group were calculated. Rates of hospitalisation and death from influenza infection were calculated and standardised by age group to the 2007 population of the Northern Territory. Plots of numbers of cases by month and proportion of each year’s cases by month and region were constructed. Case notification rates per 100 000 population and proportion of influenza A and B cases in each region were mapped. To examine which areas consistently showed higher notification rates, each area’s incidence rate was ranked, and the median rank for each area over the 10‐year period was calculated. Maps showing the ratio of the Indigenous case notification rate to the non‐Indigenous case notification rate were constructed by pooling remote SA2 areas into broad areas due to low case numbers. Only areas that contained at least 5 notifications in the broad area for a given year and a notification for an Indigenous and non‐Indigenous population member were analysed. Statistical analyses were performed using Stata version 15.1 (StataCorp).

### 2.3 Ethics approval

Ethical approval for the study was obtained from the Human Research Ethics Committee of the Northern Territory Department of Health and Menzies School of Health Research (reference number: 2017‐2894), Central Australia Human Research Ethics Committee (reference number: CA‐18‐3031), the West Australian Aboriginal Health Ethics Committee (reference number: 851) and Melbourne Health Human Research Ethics Committee (reference number: 2018.119). A waiver of consent was approved by the reviewing committees. This study was conducted in accordance with the Australian Code for the Responsible Conduct of Research.^13^

## 3 RESULTS

### 3.1 Notified cases and notification rates

Between 2007 and 2016, there were 6891 notified cases of influenza in the Northern Territory. The annual population during this period averaged 233 162. Influenza A made up of 5650 (82.0%) and influenza B 1222 (17.7%) of the cases. The remainder were co‐infections (9 cases) or untyped (10 cases). The subtype or lineage of 3756 (54.5%) of cases was known. Notification rates after 2009 were consistently higher than notification rates prior to 2009 (Table S1).

**FIGURE 1:**
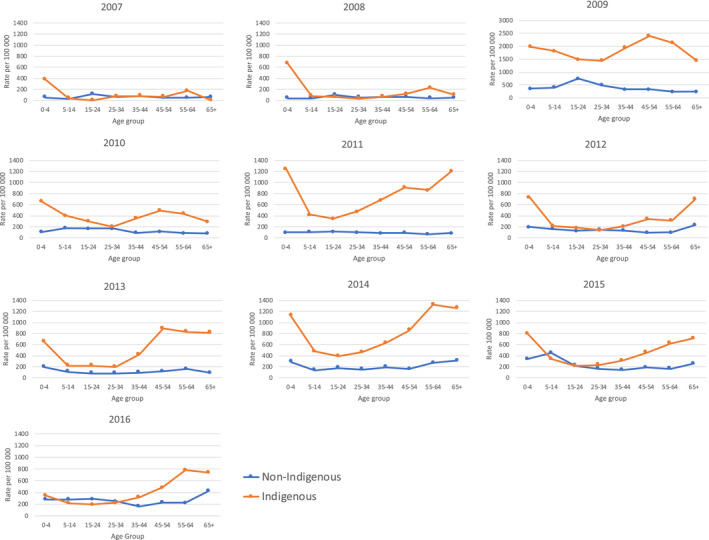
Rates of laboratory‐confirmed influenza cases per 100 000 population members in the Northern Territory from 2007 to 2016 by Indigenous status. Note case rates for 2009 presented with different scales on vertical axis

In most years and age groups, Indigenous Australians had higher notification rates than non‐Indigenous Australians (Figure 1). The greatest disparity between Indigenous and non‐Indigenous notification rates throughout the study period was for older adults, although children aged between 0 and 4 were also disproportionately affected (Table 1).

**TABLE 1:** Ratios (95% CI) of Indigenous influenza case notification rate to non‐Indigenous influenza case notification rate in the Northern Territory from 2007 to 2016 by age group. Categories marked ND indicate that there were no notifications for Indigenous or non‐Indigenous Australians that year

Age group	2007	2008	2009	2010	2011	2012	2013	2014	2015	2016	2007‐2016
0‐4	7.34 (2.85, 18.88)	16.84 (6.10, 46.49)	5.56 (3.90, 7.92)	6.11 (3.26, 11.47)	12.63 (6.76, 23.60)	3.73 (2.29, 6.08)	3.39 (2.06, 5.59)	3.86 (2.59, 5.73)	2.38 (1.59, 3.55)	1.23 (0.74, 2.07)	4.30 (3.69, 5.00)
5‐14	1.55 (0.47, 5.08)	2.22 (0.87, 5.64)	4.49 (3.49, 5.77)	2.34 (1.53, 3.58)	3.97 (2.40, 6.56)	1.36 (0.83, 2.24)	1.95 (1.13, 3.38)	3.60 (2.28, 5.66)	0.75 (0.53,1.06)	0.77 (0.50, 1.19)	2.25 (1.97, 2.56)
15‐24	ND	0.59 (0.26, 1.34)	1.97 (1.61, 2.42)	1.80 (1.16, 2.79)	3.04 (1.87, 4.94)	1.49 (0.86, 2.56)	2.85 (1.57, 5.17)	2.17 (1.43, 3.28)	0.98 (0.62, 1.55)	0.68 (0.43, 1.07)	1.58 (1.39, 1.80)
25‐34	1.20 (0.51, 2.81)	0.54 (0.16, 1.88)	2.95 (2.35, 3.71)	1.20 (0.73, 1.98)	4.71 (3.01, 7.37)	0.94 (0.53, 1.65)	2.44 (1.40, 4.26)	3.12 (2.14, 4.56)	1.47 (0.94, 2.30)	0.91 (0.60, 1.39)	2.07 (1.81, 2.36)
35‐44	1.04 (0.44, 2.45)	1.17 (0.45, 3.00)	6.04 (4.67, 7.83)	3.93 (2.32, 6.65)	8.24 (5.08, 13.38)	1.57 (0.91, 2.71)	4.58 (2.78, 7.54)	3.29 (2.27, 4.76)	2.23 (1.38, 3.60)	2.00 (1.27, 3.17)	3.71 (3.24, 4.26)
45‐54	1.46 (0.46, 4.58)	2.01 (0.81, 4.97)	7.42 (5.64, 9.75)	4.48 (2.67, 7.50)	10.20 (6.25, 16.64)	3.68 (2.07, 6.56)	7.90 (5.06, 12.35)	5.62 (3.75, 8.41)	2.50 (1.60, 3.91)	2.13 (1.42, 3.19)	4.97 (4.32, 5.71)
55‐64	3.90 (1.24, 12.30)	6.35 (2.13, 18.90)	8.88 (6.05, 13.04)	5.02 (2.48, 10.15)	13.46 (6.91, 26.21)	3.12 (1.51, 6.42)	5.28 (3.23, 8.63)	4.89 (3.36, 7.11)	3.89 (2.35, 6.46)	3.55 (2.29, 5.49)	5.56 (4.71, 6.57)
65+	ND	2.29 (0.42, 12.49)	5.95 (3.44, 10.28)	3.66 (1.27, 10.54)	14.41 (6.75, 30.74)	3.00 (1.62, 5.57)	9.12 (4.37, 19.03)	4.06 (2.56, 6.44)	2.82 (1.62, 4.90)	1.74 (1.05, 2.88)	3.79 (3.10, 4.62)

### 3.2 Notification rates by area

**FIGURE 2:**
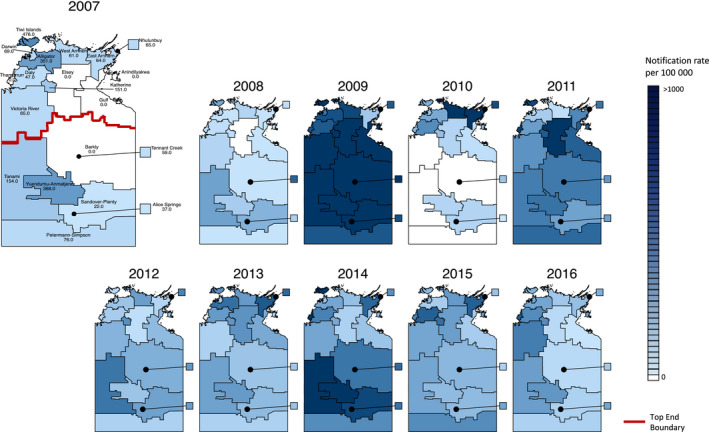
Influenza notification rates per 100 000 population in SA2 divisions of the Northern Territory, 2007‐2016. SA2 areas that were recognised to form the suburban areas of Darwin and Alice Springs were combined to form the Darwin and Alice Springs regions. The areas of Darwin, Alice Springs, Katherine, Nhulunbuy and Tennant Creek represent the major town areas in the Northern Territory. The map of 2007 was drawn larger to allow for labels to be resolved

Maps of notification rates by SA2 groupings demonstrated considerable variation by area and year (Figure 2). Tanami and the Tiwi Islands consistently ranked as having the highest rates. Notification rates in the 5 major towns of the Northern Territory (Darwin, Alice Springs, Katherine, Tennant Creek and Nhulunbuy) were broadly similar to notification rates in the surrounding remote areas.

**FIGURE 3:**
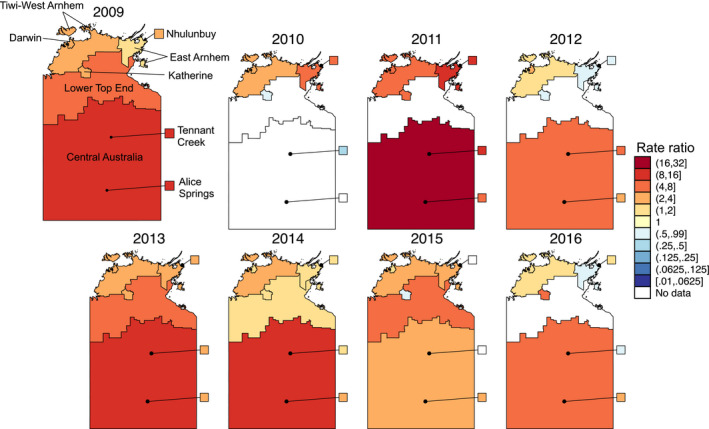
Maps of the ratio of the rate of influenza notifications in the Indigenous population to the rate of influenza notifications in the non‐Indigenous population. Areas with < 5 total notifications or no notifications in either the Indigenous or non‐Indigenous population were considered to have no valid data for this analysis. The map of 2009 was drawn larger to allow for labels to be resolved

The majority of geographical regions had higher influenza notification rates in the Indigenous population (Figure 3, Table 2). Based on raw rate ratios alone, the Central Australia region had the greatest disparity in influenza case notification rate between the Indigenous and non‐Indigenous populations. The wide confidence intervals present are likely due to small case numbers.

**TABLE 2:** Ratios (95% CI) of Indigenous influenza case notification rate to non‐Indigenous influenza case notification rate in the Northern Territory from 2009 to 2016 by area. Categories marked ND indicate that there were no notifications for Indigenous or non‐Indigenous Australians that year or <5 total notifications in the area

Area	2009	2010	2011	2012	2013	2014	2015	2016
Darwin	3.03 (2.54, 3.62)	3.04 (2.22, 4.16)	5.22 (3.48, 7.84)	2.26 (1.50, 3.40)	3.54 (2.46, 5.08)	1.72 (1.25, 2.38)	1.14 (0.82, 1.59)	1.04 (0.77, 1.41)
Alice Springs	8.56 (6.38, 11.48)	ND	4.59 (3.07, 6.87)	2.76 (1.86, 4.08)	3.87 (2.22, 6.74)	3.28 (2.42, 4.46)	2.27 (1.47, 3.52)	2.91 (1.90, 4.47)
Tennant Creek	12.8 (3.08, 53.22)	0.37 (0.07, 2.00)	9.51 (1.24,72.69)	4.39 (0.98, 19.61)	2.93 (0.33, 26.18)	1.36 (0.55, 3.32)	ND	0.95 (0.16, 5.68)
Nhulunbuy	2.68 (1.11, 6.47)	7.81 (4.10, 14.89)	8.59 (3.99, 18.52)	0.60 (0.08, 4.43)	3.26 (1.33, 8.01)	1.45 (0.48, 4.42)	ND	1.45 (0.30, 7.00)
Katherine	3.96 (2.77, 5.65)	0.77 (0.28, 2.12)	4.72 (2.93, 7.62)	0.91 (0.48, 1.73)	2.32 (1.26, 4.24)	1.92 (0.95, 3.89)	0.80 (0.47, 1.39)	4.11 (2.25, 7.48)
Central Australia	9.44 (5.04, 17.71)	ND	20.52 (2.85, 147.52)	6.18 (1.50, 25.45)	9.73 (1.34, 70.95)	9.29 (2.96, 29.18)	2.16 (0.77, 6.04)	6.26 (0.85, 45.99)
Tiwi‐West Arnhem	2.52 (1.62, 3.92)	2.42 (1.34, 4.35)	5.15 (2.07, 12.79)	1.13 (0.53, 2.43)	3.27 (1.57, 6.82)	3.05 (1.68, 5.55)	3.11 (1.34, 7.24)	1.27 (0.69, 2.35)
East Arnhem	1.26 (0.67, 2.37)	4.37 (1.61, 11.88)	10.45 (1.45, 75.46)	0.90 (0.20, 4.11)	2.27 (0.91, 5.64)	1.85 (0.67, 5.08)	2.74 (0.86, 8.72)	0.89 (0.27, 2.97)
Lower Top End	4.11 (2.18, 7.76)	ND	ND	ND	5.68 (0.77, 41.88)	1.80 (0.42, 7.77)	4.61 (0.62, 34.13)	ND

### 3.3 Rates of severe outcomes in Indigenous and non‐Indigenous Australians

Hospitalisations were 6.51 times higher (95% CI: 5.91, 7.18) amongst Indigenous Australians (with the Indigenous and non‐Indigenous rates being 237.01 and 36.40 per 100 000, respectively). Mortality was 5.46 times higher (95% CI: 2.40, 12.71) than that for non‐Indigenous Australians (with the Indigenous and non‐Indigenous rates being 3.14 and 0.57 per 100 000, respectively).

### 3.4 Variation in influenza seasonality

**FIGURE 4:**
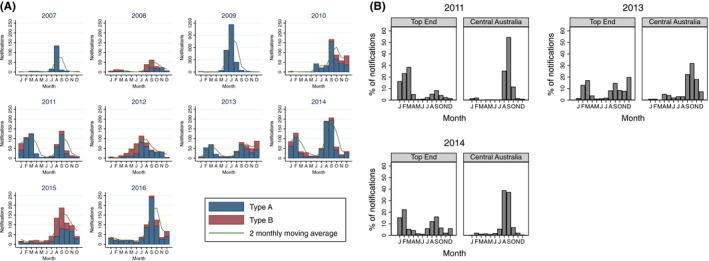
A, Laboratory‐confirmed influenza cases in the Northern Territory by month. B, Histograms of percentage of laboratory‐confirmed influenza cases seen each month in the Top End and Central Australia regions of the Northern Territory for years in which a bimodal peak in the influenza season is seen. The total number of laboratory‐confirmed influenza cases for each of these years was 2011‐Top End 447, Central Australia: 189; 2013‐Top End 407, Central Australia: 94; 2014‐Top End 543, Central Australia: 318

For most seasons, a typical Southern Hemisphere influenza epidemic curve is seen with peaks in August‐September (Figure 4A), with influenza A predominating in most time periods. However, in 2011, 2013 and 2014, an additional peak was seen during February‐March. In seasons with multiple peaks, most of the earlier cases occurred in the Top End (Figure 4B).

### 3.5 Spatial distribution of influenza A and B

**FIGURE 5:**
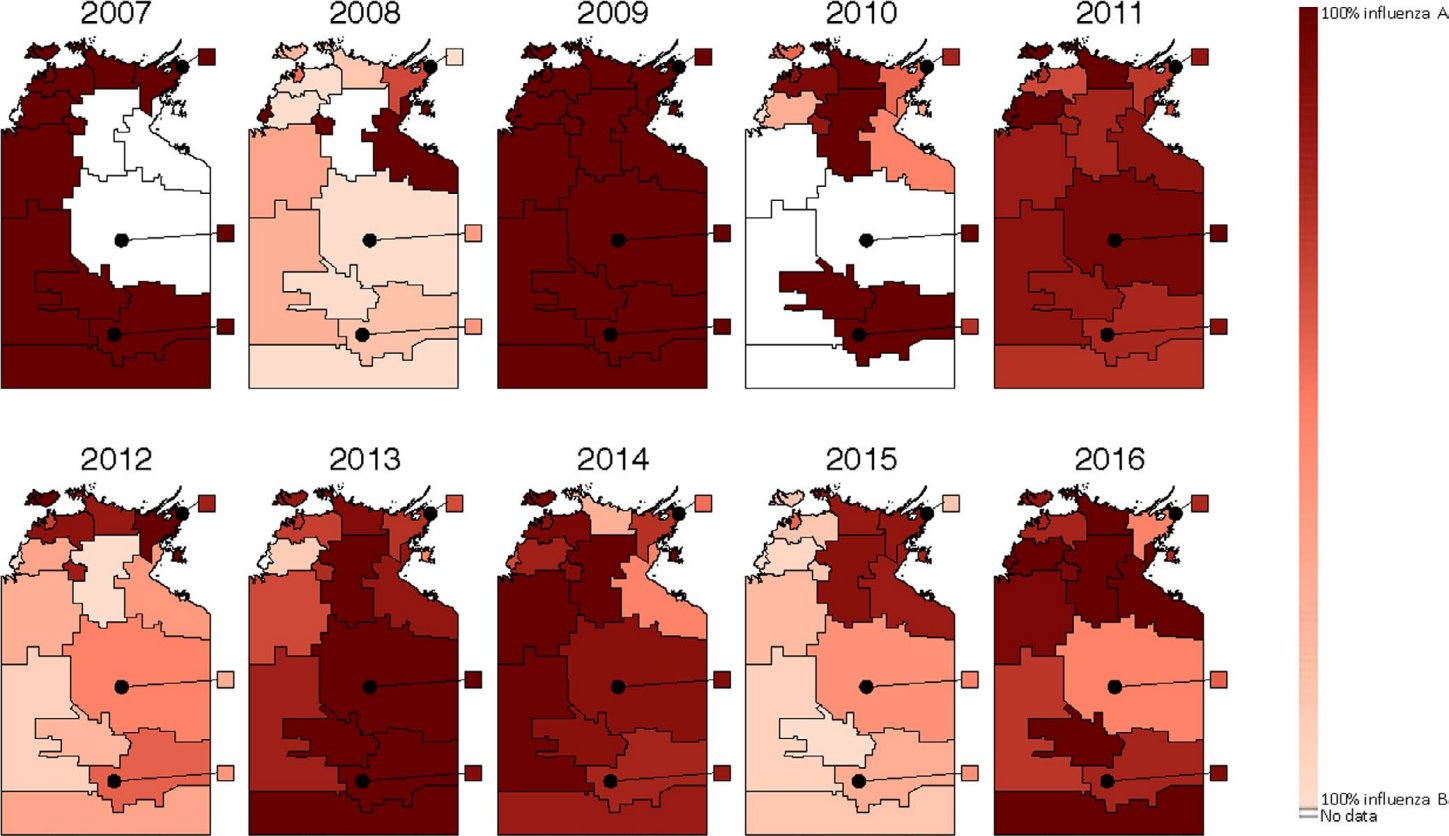
Maps of the proportion of laboratory‐confirmed influenza type A and B cases in each SA2 area of the Northern Territory from 2007 to 2016. SA2 areas that were recognised to form the suburban areas of Darwin and Alice Springs were combined to form the Darwin and Alice Springs regions. The areas of Darwin, Alice Springs, Katherine, Nhulunbuy and Tennant Creek represent the major town areas in the Northern Territory

The proportion of type A and type B cases in each SA2 area by year is shown in Figure 5. While influenza A was broadly predominant in some years (2007, 2009, 2011), for most years there was geographical heterogeneity in the predominance of influenza A and B in different regions (eg 2012 and 2015). The predominant types in the town areas are broadly reflective of the predominant type in the surrounding remote area.

## 4 DISCUSSION

Our analysis of notifications of laboratory‐confirmed influenza cases has revealed consistently higher rates of influenza cases in the Indigenous population of the Northern Territory. Influenza had a particularly disproportionate impact upon older Indigenous adults and Indigenous children aged 0‐4. We have also demonstrated that influenza outbreaks in the February‐March period in the Northern Territory are occurring in the tropical Top End.

The increased influenza rates within the Indigenous population seen in this study during both pandemic and non‐pandemic periods (Figures 1 and 3) could be associated with increased crowding within Indigenous households, which is more common in remote areas. A study which modelled an influenza‐like illness outbreak and incorporated data on household structures found that the illness would impact 90% of the population of a remote Indigenous community, 75% of an urban Indigenous community and 25% of a non‐Indigenous urban population.^14^

The higher rates of severe outcomes from infection are consistent with previous data that Indigenous populations are disproportionately affected by influenza. A study examining severe influenza A(H1N1)pdm09 cases in the Indigenous and non‐Indigenous populations of countries across the Americas and the Pacific found that rates of hospitalisations from A(H1N1)pdm09 were 3.0‐ to 7.7‐fold higher for the Indigenous population compared to the corresponding non‐Indigenous population.^15^ Furthermore, this study revealed that mortality rates from A(H1N1)pdm09 were between 3.4‐ and 5.3‐fold higher in the Indigenous population again compared to the corresponding non‐Indigenous population.^15^ Seasonal influenza too has been reported to have had a disproportionate impact upon Indigenous Australians at a national level, with studies reporting that Indigenous hospitalisation rates from influenza were 2.3‐3.9 times higher than rates for non‐Indigenous Australians.^16^, ^17^, ^18^

Genetic factors may contribute to the higher rates of severe outcomes from influenza infection in the Indigenous population.^19^ Analysis of HLA types found in Aboriginal Australians found that alleles of the A*24 type, which are thought to be less efficient at presenting conserved regions of the virus, were found at a higher frequency in Aboriginal Australians.^19^ The A*24 allele was also detected at higher frequency in Native American populations, indicating this genetic susceptibility to severe influenza outcomes is not merely restricted to Indigenous Australian populations.^19^ HLA types efficient at presenting conserved regions of the H7N9 subtype were found at lower frequencies in Aboriginal Australians compared to Caucasians, making this group more vulnerable to a subtype already known to be highly pathogenic.^20^

Comorbid medical conditions are reported to be more prominent in the Indigenous population than the non‐Indigenous population in this region,^21^ and this too may contribute to the higher rates of severe outcomes from influenza infection. For example, it was reported that 40% of Indigenous adults in the Northern Territory had chronic kidney disease compared to only 8.7% of non‐Indigenous Australians.^21^ In 2015, the prevalence of rheumatic heart disease was reported to be 37 times higher in Indigenous Australians than non‐Indigenous Australians in the Northern Territory.^21^

The data provide some guidance as to priorities for vaccination strategies. Given the consistently higher rates of notifications seen in the Indigenous population, it is important that good vaccination coverage is maintained in the Indigenous population. The extremely large difference in notification rates seen in the 0‐4 age bracket and older adults highlights the importance of maintaining high immunisation rates amongst this group. Since the change in government policy in 2015 to cover the cost of vaccination in Indigenous Australians aged 6 months to under 5 years, vaccine uptake for Indigenous Australians in the Northern Territory in this age group has increased, from <10% in 2007‐2014, to over 50% in 2015 and 2016.^22^ Although the notification rates in the 0‐4 age bracket remained higher for Indigenous Australians in 2015 and 2016, the fold difference in case rates was comparatively lower than in other years, with the fold difference in case rates in 2016 being the lowest ever seen for this age group (Table 1). This could be an early indication that the change in immunisation policy has helped reduce the disparity in influenza rates. Nevertheless, it is too soon to draw conclusions as to the effectiveness of expanding subsidised vaccination to Indigenous Australians in the 0‐4 age group based on the duration of data presented here; continued monitoring is needed. The higher notification rates seen in the Tanami region and the Tiwi Islands (Figure 2) also emphasise that maintaining high vaccination coverage in these areas is important.

The peak in influenza activity outside the typical influenza season seen in the northern Top End of the Northern Territory bears similarity to tropical countries such as Columbia, Ecuador, Costa Rica and Thailand which show 2 peaks in influenza activity.^23^

Influenza activity during the February‐March period presents challenges to effective vaccination. In the Northern Territory, influenza vaccinations are available to the public from March‐April onwards. There is growing evidence to suggest a decline in the effectiveness of the influenza vaccine over time but the rate at which the effectiveness of the vaccine declines is unclear.^24^ Nevertheless, a meta‐analysis of 14 studies revealed that significant decreases in the effectiveness of the vaccine against H3N2 and type B viruses were seen 91‐180 days after vaccination.^25^ This suggests that during outbreaks in the February‐March period, recipients of the influenza vaccine from the previous season are likely to have reduced levels of protection than during the typical influenza season. It is also possible that the strains that circulate during the February‐March period may differ from the strains that circulated during the previous epidemic peak.

The possibility of re‐vaccinating individuals against potential outbreaks in February‐March could be considered. A randomised controlled trial in Singapore found that semi‐annual vaccination led to a significant increase in the proportion of participants with antibody titres ≥1:40 for H1N1 virus only.^26^ Despite there not being an increase seen in the proportion of participants with antibody titres ≥1:40 for the H3N2 or B viruses, there may still be some clinical benefits for biannual vaccination.^26^ A lower proportion of participants that received biannual vaccination reported influenza‐like illnesses, although it was noted that the sample used in this study was too small to detect whether there was a reduction in laboratory‐confirmed influenza.^26^

Given that influenza outbreaks in the February‐March period are occurring in the Top End, a vaccination campaign against these outbreaks would be best focused on this region. However, it appears difficult to judge in which years an outbreak in February‐March would occur. It would be useful if forecasting models were developed to allow for the detection of interseasonal outbreaks, thus allowing for better informed decisions to be made on whether to revaccinate individuals in the Top End against influenza later in the year. However, recent attempts at forecasting influenza outbreaks in tropical areas have had limited success.^27^ Attempts at modelling an influenza outbreak in a tropical region suggested accurate forecasts can be developed 3 weeks in advance,^28^ but this clearly not enough time to promote and conduct a vaccination campaign in this region. Whether the predominant circulating subtype or lineage during the February‐March is the same as that during the August‐September peak also needs to be investigated.

There are some limitations associated with this data set. First, the data set is restricted to patients who present to medical care and receive a laboratory test for influenza. Since not all those infected with influenza will seek medical care and few are swabbed for influenza, the true influenza burden is underestimated. Furthermore, comparison of rates between the different regions is only valid if patients in the different regions are equally as likely to present to medical care and receive laboratory tests when presenting with influenza‐like illnesses. Laboratory testing practices have changed over the period of the study, with consistently higher rates of notifications in post‐pandemic years (Table S1). Finally, the resident location supplied with the notification is that of the patient’s home location, not where the patient presented to medical care. Thus, it is possible that a patient was infectious while present in an area different to that listed with the notification, although analysing location data by SA2 area does decrease the likelihood of misclassifying a patient’s location. It should also be noted that when examining notification rates by area, the population of many remote areas is small, and in areas where the population is small, a few cases can lead to more dramatic fluctuations in the rate than in areas where the population is large.

By analysing notifications of influenza infection in the Northern Territory, we have highlighted the unequal burden of influenza on the Indigenous population and thus the importance of maintaining good vaccination coverage, especially for those in the areas of Tanami and the Tiwi Islands. Furthermore, by demonstrating that influenza outbreaks in the Northern Territory during the February‐March period are occurring in the tropical Top End, we have established which area of the Northern Territory to target vaccination campaigns towards in order to prevent outbreaks during this period.

## CONFLICT OF INTEREST

Dr. Tong reports grants from National Health and Medical Research Council, during the conduct of the study.

## AUTHOR CONTRIBUTIONS

**Aaron Lawson Weinman:** Formal analysis (lead); Investigation (lead); Project administration (lead); Visualization (lead); Writing‐original draft (lead); Writing‐review & editing (supporting). **Sheena Sullivan:** Formal analysis (equal); Investigation (equal); Supervision (equal); Visualization (supporting); Writing‐review & editing (equal). **Dhanasekaran Vijaykrishna:** Supervision (equal); Writing‐review & editing (equal). **Peter Markey:** Data curation (lead); Resources (lead). **Avram Levy:** Resources (supporting); Writing‐review & editing (supporting). **Adrian Miller:** Supervision (supporting). **Steven Tong:** Conceptualization (lead); Formal analysis (equal); Funding acquisition (lead); Investigation (equal); Project administration (supporting); Supervision (lead); Visualization (supporting); Writing‐review & editing (lead).

## Supporting information

Table S1
