# Supplementary material for: Epidemiological trends in notified influenza cases in Australia’s Northern Territory, 2007‐2016
Source: Influenza Other Respir Viruses. 2020 May 23;14(5):541–50. doi: 10.1111/irv.12757 (PMC7431647; doi:10.1111/irv.12757)
Supplement: Supplementary file 1 — Table S1 [file IRV-14-541-s001.docx]

# Supporting Information

*Supplement 1- Annual notification rates for influenza cases in the Northern Territory, 2007-2016*

| **Year** | **Influenza notification rate per 100,000** | **95% CI** |
| --- | --- | --- |
| 2007 | 75.79 | 64.57, 88.40 |
| 2008 | 85.50 | 73.72, 98.64 |
| 2009 | 870.69 | 832.65, 910.03 |
| 2010 | 208.46 | 190.21, 227.99 |
| 2011 | 255.95 | 203.75, 277.43 |
| 2012 | 186.51 | 169.49, 204.78 |
| 2013 | 198.16 | 180.81, 216.73 |
| 2014 | 333.07 | 310.51, 356.83 |
| 2015 | 274.22 | 253.86, 295.78 |
| 2016 | 282.89 | 262.25, 304.73 |
